# Cancer cell killing by target antigen engagement with engineered complementary intracellular antibody single domains fused to pro-caspase3

**DOI:** 10.1038/s41598-019-44908-7

**Published:** 2019-06-12

**Authors:** Jennifer S. Chambers, Tim Brend, Terence H. Rabbitts

**Affiliations:** 1 Weatherall Institute of Molecular Medicine, MRC Molecular Haematology Unit, University of Oxford, Oxford, OX3 9DS UK; 2 Present Address: Leeds Institute of Medical Research at St. James’s, St James’s University Hospital, Beckett Street, Leeds, LS9 7TF UK

**Keywords:** Targeted therapies, Cancer therapy

## Abstract

Many tumour causing proteins, such as those expressed after chromosomal translocations or from point mutations, are intracellular and are not enzymes *per se* amenable to conventional drug targeting. We previously demonstrated an approach (Antibody-antigen Interaction Dependent Apoptosis (AIDA)) whereby a single anti-β-galactosidase intracellular single chain Fv antibody fragment, fused to inactive procaspase-3, induced auto-activation of caspase-3 after binding to the tetrameric β-galactosidase protein. We now demonstrate that co-expressing an anti-RAS heavy chain single VH domain, that binds to mutant RAS several thousand times more strongly than to wild type RAS, with a complementary light chain VL domain, caused programmed cell death (PCD) in mutant RAS expressing cells when each variable region is fused to procaspase-3. The effect requires binding of both anti-RAS variable region fragments and is RAS-specific, producing a tri-molecular complex that auto-activates the caspase pathway leading to cell death. AIDA can be generally applicable for any target protein inside cells by involving appropriate pairs of antigen-specific intracellular antibodies.

## Introduction

While the search for small molecule drugs to intracellular targets continues, a variety of macromolecules have been developed (we collectively refer to macromolecules of this type as macrodrugs^1^ to discriminate them from conventional drugs) that bind to intracellular target proteins and protein complexes to ablate function. For instance, antibody fragments targeting the T cell acute leukaemia protein LMO2^2–4^ or macrodrugs targeting RAS proteins that include whole antibodies^5^, antibody fragments^6^ and non-antibody formats such as DARPins^7^ have been developed. While intracellular antibodies are very effective inhibitors of protein function and of protein-protein interactions (PPIs) either as single chain Fv (scFv) or single domain formats, they require prolonged expression to sustain an anti-tumour effect, such as shown for anti-RAS intracellular antibody fragments^6,8^. This is because these macromolecules normally have no intracellular effector function to aid cell destruction, unlike normal (extracellular) antibodies that can recruit further immune system support through their constant (C) regions. In order to achieve a cell killing outcome, entities (equivalent to whole antibody effectors) must be added to the intracellular antibodies.

Several functionalities have been fused to intracellular antibodies to incorporate the equivalent of C-region effector functions when operating in cells (reviewed in^9^). In this context, induction of cell death by intracellular antibody binding is an attractive option because antibody binding would ideally induce this biological response. Apoptosis, or programmed cell death (PCD), is a normal but tightly controlled process required for cellular turnover, development and correct immune functioning^10^. Induction of apoptosis is mediated through activation of initiator caspases and completed by executioner caspases, such as caspase-3. The experimental homo-dimerization of procaspase-3 has been shown to permit self-activation and result in apoptosis of cells^11,12^. Using a model system of the naturally homo-tetrameric protein β-galactosidase and its binding by an scFv^13^, we showed that fusing the scFv to procaspase-3 resulted in antigen-dependent apoptosis due to dimerization of the scFv and auto-activation of procaspase-3^14^. The AIDA approach thus requires two adjacent binding antibody fragments to bring two procaspase-3 entities together for the auto-activation and the scFv bound to β-galactosidase achieves that because of β-galactosidase tetramerization. Normally, intacellular protein multimerization will not occur, or will not occur in a way that causes scFv dimers to locate close enough to trigger procaspase auto-activation. It was, therefore, necessary to evaluate if a trimeric antigen-antibody fragment protein-protein interaction (i.e. VH, VL and antigen) would elicit AIDA. Following optimization of intracellular expression of the domain antibodies and the subsequent development of complementary, antigen-specific VL screening methods, we found anti-RAS single light chains that could couple with a potent anti-RAS VH single domain, that binds to mutant RAS several thousand times better than to wild type RAS^6^, to form an Fv. By employing one of these VL that only binds to RAS when in the presence of the VH^15^ we were able to re-evaluate the AIDA system to develop the strategy of antigen-dependent apoptosis that requires the simultaneous binding of VH and VL-procaspase3 fusion antibodies in cells. We have used separated VH and VL from an anti-RAS scFv, to show that complementary binding of intracellular VH and VL, each fused to procaspase-3, can kill cells expressing mutant RAS. Anti-RAS VH-procaspase-3 or VL-procaspase-3 alone do not induce apoptosis. The AIDA method can therefore be adapted to kill cancer cells expressing any specific target antigen when suitable complementary pairs of variable region antibody fragments are selected.

## Results

### Induced apoptosis by targeting RAS with two domain antibody-procaspase-3 fusions

**Figure 1:**
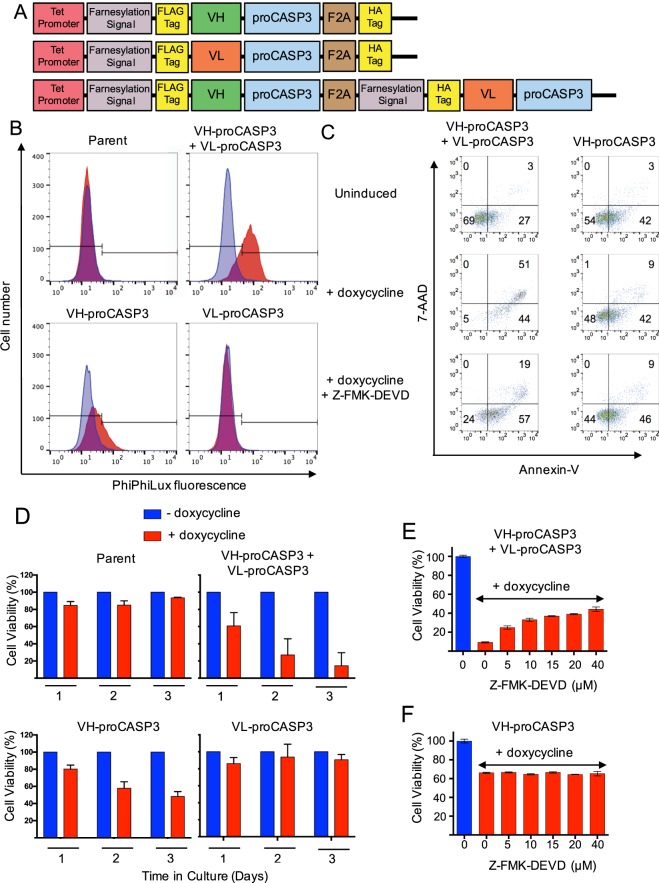
Activation of apoptosis by anti-RAS iDAb-procaspase3 fusion proteins. A schematic diagram of the AIDA expression plasmids (using the tetracycline-inducible pUHD10 plasmid) controlled by the tetracycline operator (tet-promoter) is shown in panel (A). Each expressed protein has an amino terminal farnesylation signal followed by a FLAG-tag and the iDAb (VH or VL) fused to pro-caspase-3 followed by a viral F2A sequence and a HA-tag. In the bottom line, the protein is a fusion of VH-proCASP3 and VL-proCASP3 (VH-proCASP3 + VL-proCASP3). Mutant clones were also made with mutant VH and mutant caspase-3. The activation of caspase-3 was assayed using PhiPhiLux fluorogenic substrate (panel B). Clones of each type, including the parent HT1080TetOn line, were cultured in doxycycline for 48 hours and incubated with substrate and analyzed by flow cytometry. Blue = cells without doxycycline; red = cells with doxycycline. In panel C, cells carrying VH-proCASP3 + VL-proCASP3 or VH-proCASP3 were treated with and without doxycycline, and with both doxycycline plus caspase-3 inhibitor (Z-FMK-DEVD, at 40 μM) for 48 hours and apoptotic status compared by flow cytometry with Annexin-V and 7-AAD. The numbers refer to cell numbers in each indicated quadrants. Panel D shows averaged viability analysis of three clones from each stable cell type (clones **A**–**C**) with or without doxycycline induction measured over three days using the Prestoblue (Invitrogen) resazurin-based reagent. Red bars are with and blue bars are without doxycycline. The dose response to the inhibitor, Z-FMK-DEVD, was measured over a range of concentrations to 40 μM with cells grown for 48 hours, in the presence or absence of doxycycline and with and without inhibitor. VH-proCASP3 + VL-proCASP3 cells (panel E) and VH-proCASP3 only cells (panel F). Error bars in graphs shown in panels (D–F) represent standard deviation from the mean.

We have previously identified two separate, intracellular single domain antibodies (iDAbs, one heavy chain and one light chain variable region from an scFv that was selected using Intracellular Antibody Capture (IAC)^16^ against the mammalian oncogenic protein RAS^6^. We have engineered plasmids illustrated in Fig. 1A to express each iDAb fused at the carboxy terminal end with the inactive procaspase-3. Three tetracycline-inducible plasmids were produced, either coding for the anti-VH-proCASP3 or for VL-proCASP3 or a co-expression of both VH-proCASP3 and VL-proCASP3 (herein referred to as VH-proCASP3 + VL-proCASP3). Each plasmid was stably transfected into the human fibrosarcoma cell line HT1080 (that expresses NRAS^Q61K^ mutant) and that have previously been developed to express the reverse tetracycline transactivator protein (rtTA)^8^ (these cells are referred to as HT1080TetOn or parent cells). We used the synthetic tetracycline, doxycycline, to induce iDAb-proCASP3 plasmid expression of the VH-proCASP3 and VL-proCASP3 fusions (confirmed by Western blot with anti-FLAG tag (the iDAb tag) and anti-caspase-3; Supplementary Fig. 1). The cells were induced with doxycycline for 48 hours and active caspase-3 was demonstrated by incubation with the cell permeable fluorogenic caspase-3 protease substrate PhiPhiLux (Fig. 1B). Cells in which both anti-RAS VH-proCASP3 + VL-proCASP3 were activated showed high levels of caspase-3 relative to cells only expressing VH-proCASP3 or VL-proCASP3, consistent with the active caspase-3 coming from the formation of VH-proCASP3 and VL- proCASP3 dimers at the RAS protein surface and thereby auto-activation of the dimerized procaspase-3.

Apoptosis was confirmed by flow cytometry analysis of doxycycline-induced cells using 7-aminoactinomyc in D (7-AAD, which stains the DNA of cells with damaged membranes) and annexin V detection of phosphatidylserine (following its deregulated translocation to the outer leaflet of the cell membrane during the initial stages of apoptosis). Figure 1C shows that about half of the cells expressing VH-proCASP3 + VL-proCASP3 are double positive for 7-AAD and annexin V whereas numbers in doxycycline-induced VH-proCASP3-only cells are close to those uninduced VH-proCASP3 + VL-proCASP3 cells. Treatment of doxycycline-induced VH-proCASP3 + VL-proCASP3 cells with the caspase-3 inhibitor Z-FMK-DEVD^17^ reduced the 7-AAD-annexin-V double positive population by about 60% yet little discernable effect on the profile of the doxycycline-induced VH-proCASP3-only cells (Fig. 1C). The viability of cells following iDAb expression was assessed over three days with each transfected cell type and the parent HT1080TetOn cells (Fig. 1D). Treatment of cells with doxycycline had essentially no effect on the viability of the parent cell clones nor in ones expressing VL-proCASP3 but substantial loss of viability was observed in three clones expressing VH-proCASP3 + VL-proCASP3, consistent with the RAS-VH-VL interaction activating fused procaspase-3. Clones expressing VH-proCASP3 alone show some loss of viability, consistent with previous data on the effect of the interaction of the RAS-binding iDAb on tumour growth^8^.

As validation, we further employed the caspase-3 inhibitor Z-FMK-DEVD to assess the role of the VH-procaspase-3 and VL-procaspase-3 in the cell killing by carrying out a dose response effect of caspase-3 inhibition by Z-FMK-DEVD in a clone of VH-proCASP3 + VL-proCASP3 and a clone of VH-proCASP3-only cells. The viability loss of the VH-proCASP3 + VL-proCASP3 clone was protected with increasing concentration of Z-FMK-DEVD (Fig. 1E), supporting the conclusion that these cells die because co-expressing anti-RAS VH and VL fused to procaspase-3 invokes exogenous caspase-3 activation in cells. Conversely, the VH-proCASP3-only cell viability was not affected by the inhibitor (Fig. 1F), implying that the cell killing effects are mediated by interfering with RAS-effector PPI as previously shown^6,8,18^. Morphological analysis of cells with induced expression of VH-proCASP3 plus VL- proCASP3 showed characteristics of PCD (Supplementary Fig. 2A) with cells rounding up, losing membrane definition and detaching from the culture surface within 18–24 hours while those expressing only VH-proCASP3 or VL- proCASP3 were not affected (Supplementary Fig. 2B,C respectively).

### Apoptosis depends on both VH and VL antibody fragments binding to antigen

**Figure 2:**
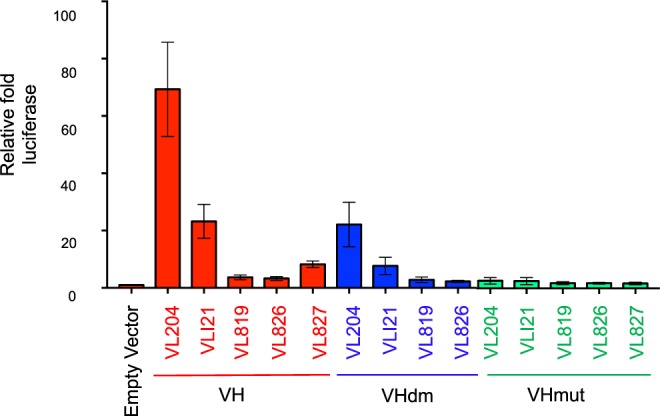
VH and VL iDAb bind to RAS in tertiary complex to induce apoptosis. Luciferase assay of HEK293T cells transiently co-transfected with plasmids encoding mutant KRAS^G12V^ antigen, various indicated VH-VP16 and VL-Gal4DBD in addition to Renilla luciferase and a Firefly luciferase reporter plasmid pG5-Luc. The expressed anti-RAS VH-VP16 fusion proteins were wild type VH, lower affinity, dematured (called VHdm) and mutant (called VHmut) and represented by red, blue and green respectively. Transfections included the anti-RAS VL (VL204) and four non-relevant VLs; VLI21 (non specific) and the anti-LMO2 VL domains VL819, VL826 and VL827. Triplicate wells were assayed 48 hours post-transfection with the Dual-Glo Luciferase reporter reagent (Promega) to assess formation of active transcription complexes. Data was analysed by calculation of the Firefly:Renilla luciferase ratio and used to derive the fold luciferase activity relative to the empty vector control. Error bars represent the standard deviation from the mean.

Our data show that co-expressing anti-RAS VH- and VL- linked to procaspase-3 results in cell death by apoptosis. The anti-RAS antibody fragment, RAS antigen and exogenous caspase-3 dependency was confirmed using modified forms of the expressed proteins. A luciferase reporter assay was devised to ensure that AIDA requires both VH-proCASP3 and in VL-proCASP3 binding RAS and is not due to antigen-independent interaction between the β-strands of the VH and VL^19^. HEK293T cells transfected with G5-luc (a Firefly luciferase (Fluc) reporter with GAL4 DNA binding sites (DBS) and vectors expressing KRAS^G12V^ and the anti-RAS iDAb VH, or mutant VH, fused to VP16 transactivation domain and various VL single domains, fused to the GAL4 DNA binding domain (DBD). When anti-RAS VH was co-expressed with the complementary VL204^6^, we observed transcriptional activation of Fluc (Fig. 2; Supplementary Fig. 3). Similar Fluc activation was found with VLI21^20^ but to a lesser extent. No significant activation was observed when we expressed non-relevant VL, that had been selected with LMO2 protein^15^. The importance of the anti-RAS VH was corroborated using a two VH mutants. In one mutant, VH complementarity determining region (CDR) mutations resulting in reduced affinity (dematured version, designated VHdm) and the second, a mutant (designated VHmut) that no longer binds KRAS^6^. (The CDR sequences of the three forms of the anti-RAS iDAb are shown in Supplementary Table 1). In the luciferase assay, VHdm retains binding to RAS when co-expressed with VL204 whilst VHmut has no ability to stimulate luciferase (Fig. 2). These data confirm that the tripartite interaction of anti-RAS VH and a complementary VL (such as VL204) with RAS antigen in this cell system is necessary for luciferase activation.

**Figure 3:**
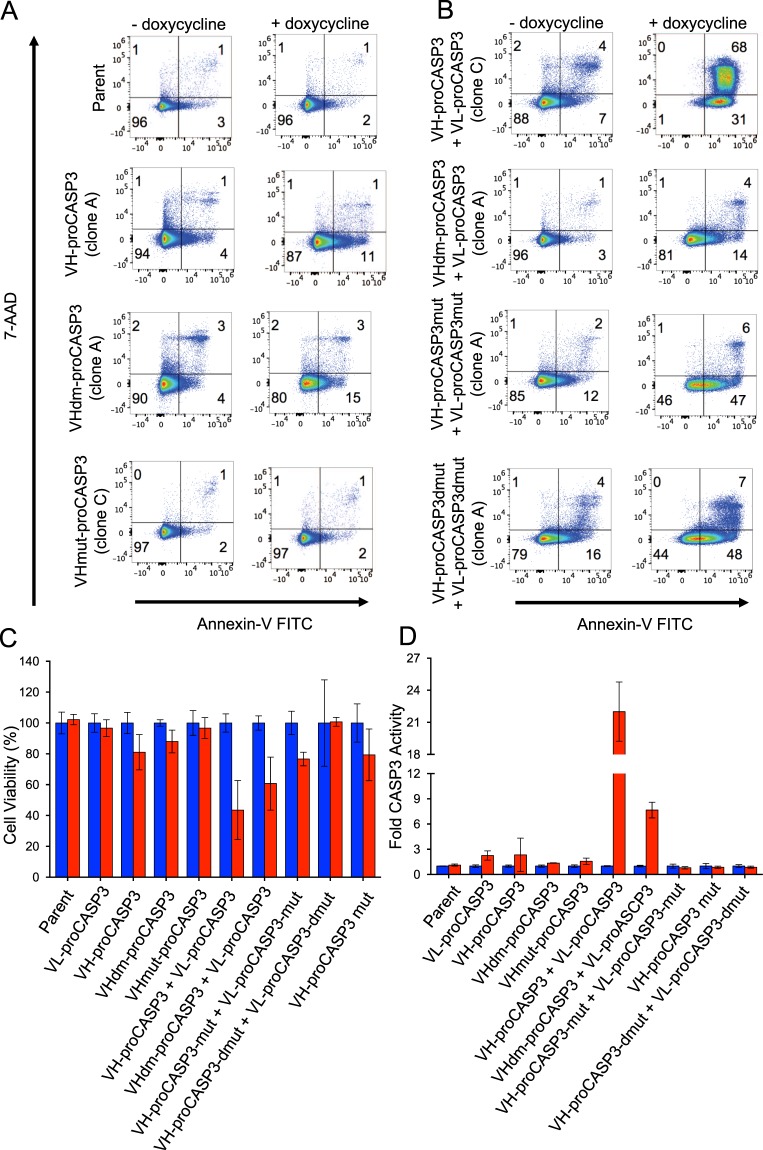
Effect of mutations of VH and caspase-3 on AIDA response. One clone of each type was grown for 48 hours, with and without doxycycline and analyzed for induction of apoptosis by flow cytometry with Annexin-V (FITC) and 7-AAD (Panels A,B). Panel (A) shows the effect of the VH mutations in the single vectors, whilst panel B shows the effect of VH and CASP3 mutations is to reduce induction of apoptosis in VH-proCASP3 + VL-proCASP3 expressing cells. The viability (panel C) and caspase-3 activity status (panel D) of these clones was judged after 48 hours using the ApoToxGlo (Promega) combined assay in the presence (red bars) or absence (blue bars) of doxycycline. Viability measurements were averaged and converted to a percentage value for comparison between clone types. Caspase-3 activation was determined by the increase in luminescence following cleavage of a reporter peptide. Data were converted to fold activation value, with no doxycycline readings given a nominal value of 1 to allow comparison between clone types. Error bars on graphs in C and D represent standard deviation from the mean.

The importance of the VH-RAS interaction on the AIDA was further established using the mutant VHs in flow cytometry of annexin-V and 7-AAD staining. While uninduced cells show little annexin-V or 7-AAD staining (Fig. 3A,B, LH panels), induction of VH-proCASP3 + VL-proCASP3 resulted in about two thirds of cells displaying both annexin-V and 7-AAD stain and about one third displaying annexin-V only (Fig. 3B, upper RH panel). Little apoptosis was seen in cells with induced VH-proCASP3-only or with the dematured VH version (VHdm-proCASP3, Fig. 3A, middle two RH panels) or with mutant anti-RAS VH (VHmut-proCASP3-only (Fig. 3A lower RH panel). Determining the viability of cultures and levels of active pro-caspase-3 at the same time after induction (48 hours) showed that those expressing VH-proCASP3 + VL-proCASP3 were about 50% viable (Fig. 3C) and had about 25-fold greater pro-caspase-3 levels than the other induced cell lines (Fig. 3D). Mutating the procaspase-3 (introducing catalytic residue mutations^21^ as single Cys163, designated (CP3mut) or double (Cys163 and His121, designated CP3dmut) into the procaspase-3 domains ablated the apoptosis (Fig. 3B lower two RH panels).

**Figure 4:**
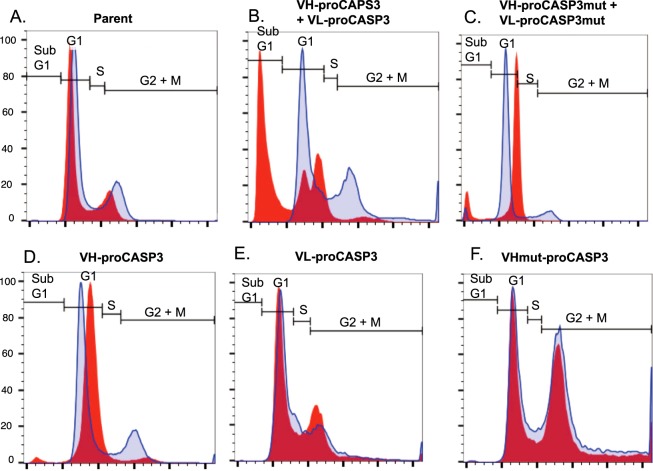
Effect of the AIDA on cell cycle progression. The effect of expressing the VH/VL-proCASP3 proteins and their variants on the cell cycle was assessed by flow cytometry. Cells were induced for 48 hours, fixed, permeabilized, and stained with 7-AAD. Live, single cells were gated according to their size and granularity by forward and side scatter parameters. The normal cell cycle populations (G0/G1, S and G2/M phases) were set on the uninduced Parental cell line. Data are represented as overlaid histograms, with the cell cycle gates shown on each plot. The regions for subG1, G1, S and G2 + M phases are indicated. Blue trace = uninduced cells Red trace = tetracycline induced cells.

A hallmark of CASP3 activation is the digestion of genomic DNA at inter-nucleosomal sites. This results from CASP3 cleavage of the inhibitor of caspase-activated DNase (ICAD), allowing the caspase-activated DNase (CAD) activity to degrade chromosomal DNA. Cell cycle analysis by flow cytometry provides evidence for the occurrence of CAD-mediated DNA degradation in the form of a sub-G1 population that is detectable as events occurring below the G1 band when cellular DNA is stained with 7-AAD. Analysis of the cell cycle in the VH-proCASP3 + VL-proCASP3 induced cells compared to control cell lines was carried out using flow cytometry (Fig. 4). When we compared the status of parent cells with and without doxycycline induction, we observed very little difference (panel A). Furthermore uninduced VH-proCASP3 + VL-proCASP3 cells showed a cell cycle distribution comparable to the parent cells (panel B). However, induction of the VH-proCASP3 and the VL-proCASP3 proteins resulted in substantial levels of sub-G1 cells (about 45%). The accumulation of the sub-G1 population was ablated in cells induced to express the VH- and VL-fused to mutant proCASP3 (panel C), confirming that the apoptosis results from exogenous caspase-3 and RAS antigen binding. A sub-G1 population was not observed within induced cells expressing either VH-proCASP3 only (panel D), VL-proCASP3 only (panel E) or mutated (non-binding) VH-proCASP3 only (panel F). Therefore, the sub-G1 cells occurring from induced apoptosis (AIDA) is VH and VL dependent and exogenous caspase-3 dependent, as well as RAS antigen dependent.

### AIDA causes RAS-antigen dependent cytotoxicity

**Figure 5:**
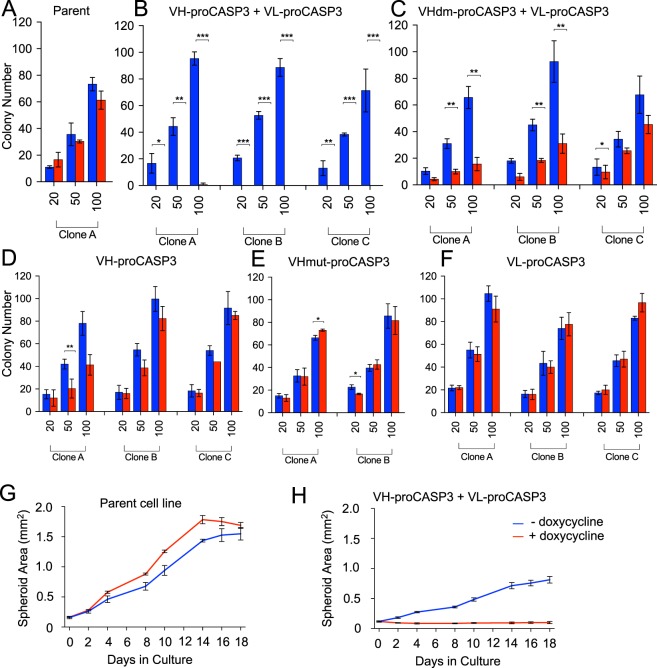
Cytotoxicity caused by VH-VL-procaspase3 using *in vitro* tumorigenicity models. Two types of *in vitro* assays were used to follow cytoxicity following VH-proCASP3 + VL-proCASP3 induction, namely a minimal dilution plating clonogenic assay (panels A–F) and a 3-D spheroid assay (panels G,H). In the plating assay, up to three clones of each type clones indicated (clones **A**–**C**) were plated at 20, 50 or 100 cells per dish (in triplicate) and grown for 9 days with and without doxycycline. Cell were fixed and stained with crystal violet for visualization. Colonies were graded as foci if they contained ≥10 cells. The bar charts show the averaged colony counts for each cell type in the presence of doxycycline (red bars) or absence (blue bars) of doxycycline. Panel A. Parental HT1080TetOn line; Panel (B). VH-proCASP3 + VL-proCASP3 clones (**A**–**C**); Panel (C). dematured VH VHdm-proCASP3 + VL-proCASP3 clones (A–**C**); Panel (D). VH-proCASP3 clones (**A**–**C)**; Panel E. mutant VH VHmut-proCASP3 clones (**A**,**B**); Panel (F). VL-proCASP3 clones (**A**–**C**). Statistical significance of the data was determined by performing Student T-tests, where significant p values (<0.05) are indicated by the following key; *<0.05, **≤0.01 and ***≤0.001, where n = 3 readings per condition. Error bars represent standard deviation from the mean. The growth in spheroid development was measured over an 18 day period comparing the parent HT1080TetOn line (panel G) with the VH-proCASP3 + VL-proCASP3 clone (panel H). Images were taken and area measurements were analyzed by FIJI software^34^. Error bars represent standard deviation from the mean, with n = 4 for all conditions.

Since our purpose was to design a method for cytotoxicity in tumour cells by dual binding of iDAb fused to inactive caspase-3, we modelled cell killing in 2-D and 3-D culture systems. In a cloning assay, cells were plated in three different densities (the plating efficiency of the different cell types is shown in Supplementary Fig. 4A) and cultured with or without doxycycline for nine days before colony counting. Colony formation in doxycycline-treated parent cells was nearly 100% compared untreated cultures (Fig. 5A) whereas there was a near total absence of colonies with induced VH-proCASP3 + VL-proCASP3, compared to those uninduced (Fig. 5B). Dematuration of the anti-RAS VH in VHdm-proCASP3 + VL-proCASP3 cells showed reduced colony formation compared to the parental control, consistent with the lower affinity with which the dematured VH binds to KRAS (Fig. 5C). Further, there was very little difference between induced and uninduced cells expressing VH-proCASP3 alone (D), non-binding mutant VHmut-proCASP3 (E) or expressing VL-proCASP3 alone (F).

As an alternative assay for cytotoxicity we established a 3-D spheroid colony assay. Tumour spheroids formed from the parental line and one VH-proCASP3 + VL-proCASP3 clone were supported in matrigel and expanded to approximately 0.5 mm before being grown with or without doxycycline for 18 days and assessed for changes in size and viability. The parental cells showed similar kinetics in the presence or absence of doxycycline (Fig. 5G). By contrast, the doxycycline induced VH-proCASP3 + VL-proCASP3 spheroids did not grow at all (Fig. 5H) while the uninduced VH-proCASP3 + VL-proCASP3 spheroids continued to grow. The average viability of VH-proCASP3 + VL-proCASP3 spheroids remaining after doxycycline-induced expression of the AIDA constructs were minimal (with fluorescence readings barely above background) compared with that of the non-doxycycline treated cells (Supplementary Fig. 4B). The cytotoxicity only occurs in the dual VH and VL expressing cells.

## Discussion

### AIDA can convert cytostatic macrodrugs to cytotoxic ones

Our previous work showed that scFv linked to procaspase-3 would self-activate caspase-3 when binding to a tetrameric target protein^14^. We now show that interaction of separated VH and VL, each fused to procaspase-3, will incur a similar self-activation caspase-3. We used NRAS as our test target antigen and apoptosis was induced by VH and VL, each fused to procaspase-3 while the individual VH- procaspase-3 or VL- procaspase-3 fusion did not cause apoptosis. Therefore the cell death depends on an antigen interaction by both V-region domains fused to procaspase-3. The dimerization of RAS protein in cells occurs at the plasma membrane^22^ but we found no evidence that the single VH or VL-procaspase-3 protein cause cell death. This is presumably because the spatial arrangement of a potential quadrameric complex of RAS-iDAb-procaspase-3/RAS-iDAb-procaspase-3 precludes procaspase-3 dimerization.

The use of the RAS intracellular antibody system was implemented here to show that AIDA can be performed with separate VH and VL to yield a ternary complex of antigen, VH and VL wherein the antigen dimerization status is not problematic. Could anti-RAS AIDA be therapeutically useful given the ubiquitous expression of RAS proteins? In initial experiments using the anti-RAS intracellular antibody, we found that it halted tumour growth in xenografted human cancer tumours^8^. When intracellular antibody induction was stopped, tumour growth re-started. Addition of inactive procaspase-3 to anti-RAS VH and VL converts binding into a cytotoxic effect. Even though the intracellular antibody binds to mutant RAS to a much greater efficiency in cells than to wild type RAS^6^, it would be mandatory for *in vivo* use to have an exquisitely tumour-specific delivery option and that does not exist as yet.

### AIDA technology can be adapted to any target in disease cells

This method can be adapted to any target protein inside cells and can be extended to any internal antigen for which two intracellular antibody fragments can be selected or are available with complementary binding sites on the antigen. Thus, it could be widely applicable to any tumour-specific protein, including chromosomal translocation fusion protein products where iDAbs binding near each other, but traversing the fusion junction could be selected. It may also be possible to adapt the method to target two members of a protein complex.

The AIDA method could also be altered for use with other intracellular macrodrugs including DARPins^23^ and peptide aptamers^24^. Further, while we have used a VH and VL pair in this concept study, it is possible to adapt to VH-VH or VL-VL pairs. The key to the functionality of this tertiary system is antigen-specificity of the binding partners and antigen-dependent caspase activation. In order to maximize optimization, further adaptations could be developing engineered procaspase-3 domains with decreased potency^25^. Further, it is important that intrinsic non-antigen-associated interaction of VH and VL does not cause procaspase-3 activation *per se*. With this in mind, in future it may be beneficial to engineer framework mutations into the VH and VL sequences to minimize any untoward macrodrug self-activation.

Macrodrugs, such as the engineered iDAbs needed for AIDA, will not enter cells *per se* because they are proteins. For the use of macrodrugs in general, methods for delivering them to cells *in vivo* are being developed. Recent work shows promise for cell targeting and delivery technologies^26^ and using mRNA for expression of proteins has been described^27,28^. We have developed a new lipid nanoparticle (LNP_LH_) that can encapsulate mRNA and deliver to a variety of cells^29^ that can potentially be developed into a delivery vehicle for cancers to facilitate the co-expression the iDAb-proCASP3 proteins. It will be important in addition to confer cell-selectivity on the delivery vehicles to ensure normal cells are not influenced by the AIDA reagents if the target protein is present. Application of these technologies should bring the AIDA technology into clinical use.

## Materials and Methods

### Vector construction

A Hygromycin resistance cassette under control of a PGK promoter was inserted into pUHD10-3 plasmid to create pUHD10-3H. Insertion of synthesized oligos (Sigma) created a modified multiple cloning site for downstream cloning, and subsequently FLAG and HA tags and the F2A site were amplified by PCR using Phusion polymerase (New England BioLabs) and ligated. VH- (#Y6)^30^ and variants, and VL- (#204)^15^ procaspase-3 fusions were also amplified PCR amplified and cloned either singly or sequentially in the case of the co-expressing VH and VL vector. Later an N-terminal farnesylation signal derived from Neuromodulin/GAP43 was added to create membrane-targeted constructs. Procaspase-3 catalytic mutations (C163S and H121G) were introduced by PCR mutagenesis, and subsequently sub-cloned into the vectors.

### Cell culture and DNA transfection

HT1080 cells expressing the reverse Tetracycline transactivator^31^ (referred to as HT1080TetOn or Parental) were cultured in DMEM with Glutamax (Gibco) and supplemented with 10% FBS (Sigma) and Penicillin/Streptomycin (Gibco) and incubated at 37 °C, 5% CO_2_. 4 × 10^5^ Cells were transfected in one well of a 6-well plate, with 3 μg of SpeI-linearized plasmid and 10 μl Lipofectamine 2000 (Invitrogen) in a total volume of 1 ml Optimem (Gibco) for 3–5 hrs. Medium was replaced and cells split at 48 hrs post-transfection and replated onto 10 cm dishes. Antibiotic selection was applied 72 hrs post-transfection, with 500 μg/ml G418 (Gibco) and 200 μg/ml Hygromycin B (Invitrogen). Selective medium was replaced every two days for approximately 7–12 days, until the control (untransfected) plate was devoid of cells. Resulting colonies were picked, propagated and screened by Western blot. Induction of doxycycline-inducible transgene expression was performed with 1 μg/ml doxycycline Hyclate (Sigma) on cells plated at an initial density of 2 × 10^4^/cm^2^.

### Western blot analysis

After 48 hrs of doxycycline induction, 10 μg total protein was boiled for 5 mins in Laemmli buffer (125 mM Tris pH6.8, 5% β-Mercaptoethanol, 2% SDS, 10% Glycerol, 0.002% Bromophenol Blue). Samples were loaded per lane onto SDS-PAGE gels alongside 3 μl BluEye Prestained protein ladder (GeneFlow). Gels were transferred to PVDF membrane (GE Healthcare) at 350 mA for 1 hr. Membranes were blocked in TBS-T buffer (1x TBS (Severn Biotech), 0.1% v/v Tween-20 (Sigma)) containing 5% non-fat milk powder for 1 hr at room temperature. Primary antibodies were incubated in TBS-T with 1% milk as follows; M2 anti-FLAG (Sigma), anti-CASP3 and anti-HA tag (both Cell Signaling), were used at 1:1000 dilution, β-Actin (Sigma) at 1:4000 dilution, with all incubated at 4 °C overnight. Secondary HRP-linked antibodies (anti-mouse and anti-rabbit, both Cell Signaling) diluted 1:1000 in TBS-T with 1% milk were applied for 1 hr at room temp. Blots were incubated with ECL reagent (Pierce) and developed on film (Sigma) with X-O-Graph developer system.

### PhiPhiLux CASP3 activity

At 48 hrs post-doxycycline induction, 5 × 10^5^ cells per growth condition were pelleted at 200 × g, 5 min at room temperature and resuspended in at total volume of 83 μl (comprising 75 μl PhiPhiLux reagent and 8 μl FBS), and incubated for 30 min at 37 °C. Cells were washed in 1 ml PhiPhiLux buffer and pelleted as before. Cells were resuspended in buffer and fluorescence measured by flow cytometry on the FITC channel of a Cyan ADP analyser (Beckman Coulter). Data was analyzed using FlowJo software (TreeStar).

### Annexin V and 7-AAD flow cytometry analysis

At 48 hrs post-doxycycline induction, cells were harvested and pelleted at 200 × g for 5 mins, room temperature. 5 × 10^4^ cells were washed and resuspended in 100 μl in Annexin V buffer (10 mM HEPES pH7.4, 140 mM NaCl, 2.5 mM CaCl_2_) with 2 μl Annexin V-FITC (BD Pharmingen) and stained on ice for 30 mins. Cells were washed and resuspended as before, with 5 μl 7-AAD (50 μg/ml, BD Pharmingen) and incubated on ice for 15–20 mins diluted with annexin V buffer immediately before collecting fluorescence measurements on an Attune flow cytometer (Invitrogen). Data was analyzed using FlowJo software (TreeStar).

### Cell cycle analysis

Cell clones were plated in 6-well plates at an initial density of 2 × 10^4^/cm^2^ in two triplicate sets (for −/+tetracycline) and left to adhere overnight. Growth medium was replaced the following day, and induction was performed with 1 μg/ml tetracycline (Sigma Aldrich, Dorset, UK). After 48 hrs cells were harvested with 0.05% Trypsin (Gibco, MA, USA) and pelleted at 200 × g for 5 mins at room temperature. 5 × 10^4^ cells were washed in PBS, fixed and permeabilized in 2 ml of ice-cold 70% ethanol followed by incubation at −20 °C for at least one hour. Cells were pelleted as before, washed twice in PBS and resuspended in 50 μl of 50 μg/ml 7-AAD staining solution (BD, New Jersey, USA), supplemented with 0.1% Triton X-100 (Sigma Aldrich, Dorset, UK), 2.5 units of RNase A (Thermo Scientific, MA, USA). Cellular DNA was stained for 30 mins at room temperature while protected from light. Samples were diluted with PBS immediately before collecting area fluorescence measurements on a linear scale with an Attune flow cytometer (Invitrogen, Paisley, UK). Data were analyzed using FlowJo software (TreeStar, OR, USA) where cell cycle gates (Sub G1, G1, S and G2/M phases) were set on the uninduced (no tetracycline) sample of each clone.

### Viability assays

Cells were plated in triplicate at 4 × 10^4^ per well in 24-well culture plates, and protein expression was induced with doxycycline as before, for 48 hrs where appropriate. PrestoBlue Viability reagent (Invitrogen) was diluted 1:10 in normal growth medium and applied to cells for 20 mins at 37 °C, 5% CO_2_. Fluorescence was measured at 544 nm excitation and 590 nm emission with a Spectramax M2e (Molecular Devices) plate reader. Blank (no cell) well readings were averaged and subtracted from all experimental data points. Results were converted to a percentage viability relative to the respective clone uninduced reading, to allow comparison between clone types.

### Caspase-3 inhibitor experiments

Cells were plated in triplicate as before in 24-well culture plates, and protein expression was induced with doxycycline for 48 hrs where appropriate. Irreversible CASP3 inhibitor Z-DEVD-FMK (Santa Cruz Biotechnology) was reconstituted in DMSO (Sigma) to 20 mM and applied to cells over a range of concentrations from 0–40 μM with standard addition of doxycycline. Controls consisted of i) uninduced, no inhibitor and ii) induced, no inhibitor wells. All wells were normalized to contain 0.2% v/v DMSO. Data was collected and analyzed by Annexin V, 7-AAD flow cytometry and viability assays.

### Spheroid growth

The method adapted from Cultrex 3-D Spheroid Fluorometric Proliferation/Viability assay (Trevigen)^32,33^. Cells were harvested with 0.05% Trypsin (Gibco, MA, USA) and pelleted at 200 × g for 5 mins, at room temperature. After counting, cells were kept on ice to ensure they were chilled ready for matrigel addition. Cells were washed once in PBS (Gibco), and resuspended in a 1:10 mix of ice-cold matrigel (BD, New Jersey, USA) to growth medium at a density of 2 × 10^4^/ml. 50 μl of cell mixture was added to each well of a ultra-low adhesion round-bottom 96-well plate (Corning, New York, USA) and centrifuged at 200 × g, 5 mins, 4 °C. Plates were transferred to a 37 °C, 5% CO_2_ incubator for 30 mins to allow the matrigel layer to set. Once solidified, 50 μl of growth medium was overlaid, with those wells receiving doxycycline beginning treatment when spheroids reached ~0.5 mm diameter. Induction of doxycycline-inducible transgene expression was performed with 1 μg/ml tetracycline (Sigma Aldrich, Dorset, UK). Plates were cultured for the next 14 days, with light microscope images taken every two days. Extra growth medium was added at regular intervals to avoid accidental damage to the spheroids. At the end of the experiment, area changes were calculated using FIJI image analysis software^34^ and spheroid viability was measured using PrestoBlue reagent (Invitrogen, Paisley, UK) as previously described. Unpaired two-tailed student t tests were performed on the viability assay results and calculated by GraphPad Prism 7.0 software (GraphPad Software, CA, USA).

### ApoToxGlo (Promega) assay

Cells plated in 96-well plates at an initial density of 2 × 10^4^/cm^2^ with doxycycline induction was performed as before. Etoposide was added to control wells at 100 μM for 8 hrs. Assay was measured at 18 and 48 hrs post-Tet addition and performed according to the manufacturer’s instructions. Fluorescence and luminescence measurements read by a M2e Spectramax plate reader (Molecular Devices). Averaged blank well readings were subtracted from all data points. Results were converted to either percentage (Viability) or fold increase (CASP3) relative to the respective clone’s un-induced reading.

### Clonal assay

The method was adapted from Franken *et al*.^35^. Cells were harvested and pelleted in a bench top centrifuge (Eppendorf) at 200 × g for 5 minutes at RT. The supernatant was discarded and cells were resuspended in 10 ml of growth medium and viable cells were counted by trypan blue dye exclusion. A working cell suspension of 2 × 10^2^ cells/ml for plating was prepared by serial dilution. Appropriate volumes of the working suspension were mixed with medium to achieve (i) 20, (ii) 50 and (iii) 100 cells in a total volume of 4 ml per 6 cm diameter plate (Corning). Six replica plates were made per cell density to allow triplicate plates of both growth conditions (+/−doxycycline). Next day, medium was removed and replaced with either fresh medium (three plates) or medium containing 1 μg/ml doxycycline (three plates). Cells were incubated at 37 °C, 5% CO_2_ for the next nine days, replacing medium every three days (+/−doxycycline). On day nine, the growth medium was removed and the cell layer washed with PBS (Gibco). Colonies were stained for 30 minutes with 3 ml staining solution (6% v/v glutaraldehyde (Sigma Aldrich), 0.5% w/v Crystal Violet (Sigma Aldrich)). Plates were washed twice with deionized water and finally gently submerged in deionized water to remove the last traces of dye, and left to dry. Cell patches of greater than 10 cells were deemed to be a colony for this assay (due to the anticipated growth impedance effect of the anti-Ras VH). Colonies were visualized on a large-field view stereomicroscope (Leica MZFLIII) and counted using a colony counting pen (VWR). The Plating Efficiency (PE) of each clone was calculated from combined average colony counts of all cell densities tested of the uninduced (no doxycycline) plates.

### Luciferase assays

Combinations of VH and VL variants (anti-RAS VH, VHdm and VHmut, anti-RAS VL#204, non-specific VL#I21 and anti-LMO2 VLs #819, #826 and #827^15^) were cloned into the triplex vector^16^ via NotI/SfiI and BamHI/EcoRV restriction sites. HEK293T cells were transiently co-transfected in triplicate with the 400 ng triplex plasmids encoding the various combinations of both VH-VP16 and VL-Gal4DBD, alongside 200 ng pG5-Luc reporter and 200 ng pPGK-KRAS_166_(G12V)-2A-Puro RAS antigen plasmid. Cells were analyzed for luciferase activity 48 hours post-transfection using the Dual-Glo Luciferase assay (Promega), with luminescence measurements read by a M2e Spectramax plate reader (Molecular Devices). The ratio of Renilla to Firefly luciferase was calculated. Fold luciferase activity was calculated relative to the empty vector control.

## Supplementary information

Supplementary data files
