# Supplementary material for: Cancer cell killing by target antigen engagement with engineered complementary intracellular antibody single domains fused to pro-caspase3
Source: Sci Rep. 2019 Jun 12;9:8553. doi: 10.1038/s41598-019-44908-7 (PMC6561968; doi:10.1038/s41598-019-44908-7)
Supplement: Supplementary file 1 — Supplementary data files [file 41598_2019_44908_MOESM1_ESM.pdf]

Supplementary Information

Cancer cell killing by target antigen engagement with engineered complementary intracellular antibody single domains fused to pro-caspase3

Jennifer S. Chambers, Tim Brend & Terence H. Rabbitts [*]

Table S1

**VH designation**	**CDR status**	**CDR1**	**CDR2**	**CDR3**
Y6	original	GFTFSTFSMN	YISRTSKTIYYADSVKG	GRFFDY
Y6dm	dematured	GFAFAAFSMN	YISRTSKTIYYADSVKG	GAAFDY
Y6mut	mutant	GFTFSRPQMA	YISRTSKTIYYADSVKG	GRFFDY

Supplementary Table 1. Anti-RAS iDAb VH variant sequences
The CDR sequences of the anti-RAS VHY6 wild type and mutant are shown, with amino acid residue substitutions highlighted in red.

Figure S1

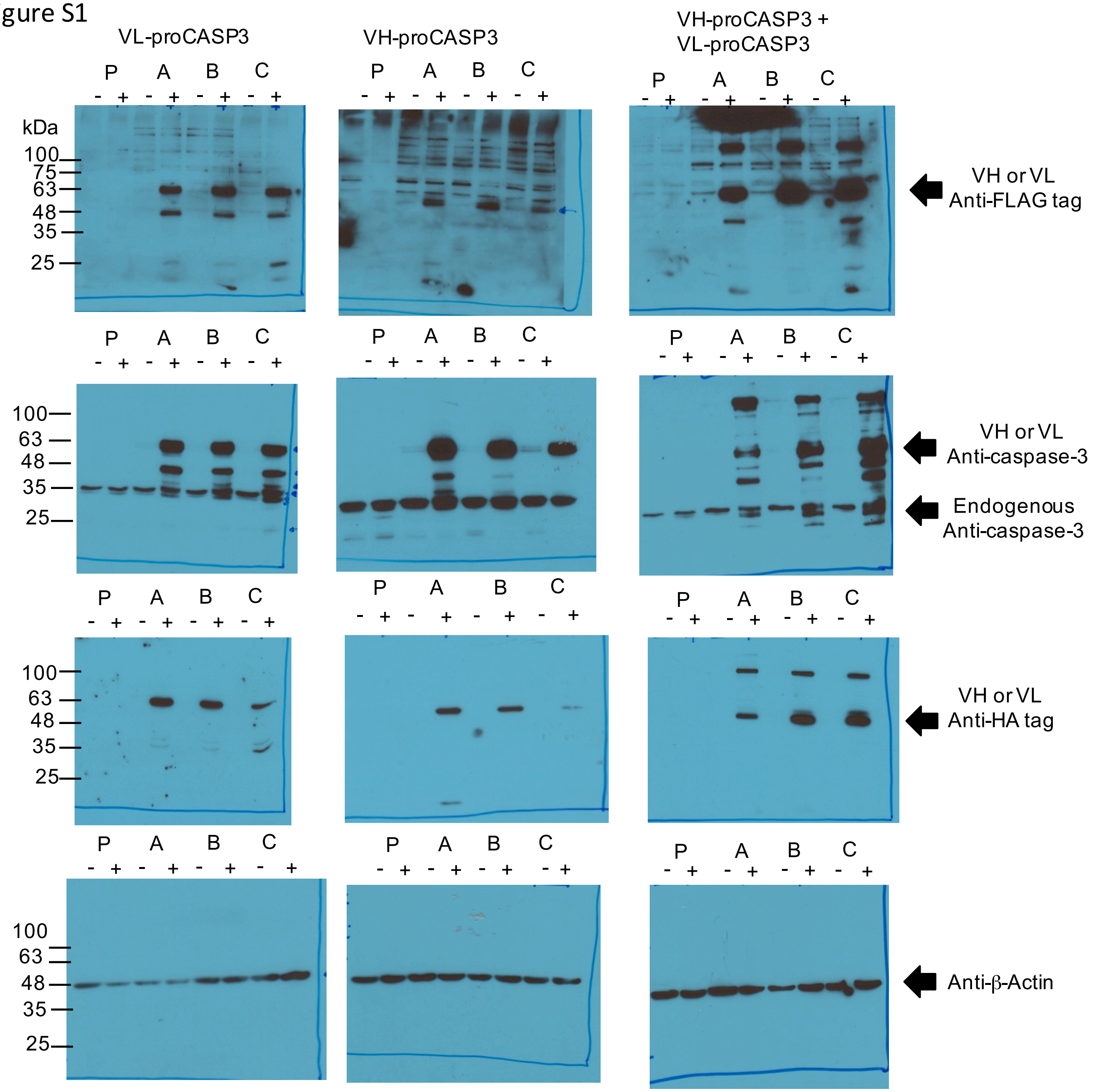

Supplementary Figure 1. Western blot analysis of VH-proCASP3 and VL-proCASP3 expression at 48hrs +/- Tetracycline induction.

Three clones of each transfected HT1080 cell (clones A, B, C) were chosen and taken forward for subsequent testing. Cell lysates from doxycycline induced (+) or uninduced (-) cells were separated by SDS-PAGE and transferred to separate membranes. The various proteins were detected using western detection method with VH proteins detected by anti-FLAG antibody, VH or VL-procaspase3 by anti-caspase 3 antibody and VH or VL with anti-HA tag antibody. Anti-b-actin for detecting b-action was used as a loading control. Protein size markers are shown on the LHS of the gels. P = lysate from un-transfected parental cells. Each SDS-PAGE separation was blotted to membrane and the complete membrane signal is shown. We have indicated by arrows the expressed proteins found in whole cell extracts in the western blots. The multiple products found in the analysis of the VH- and VL-procaspase3 proteins are due to AIDA activated apoptosis (the smaller bands), whilst larger bands are due to high molecular fusion proteins expressed after the inefficiency of the ribosome skipping at the 2A peptide.

Figure S2

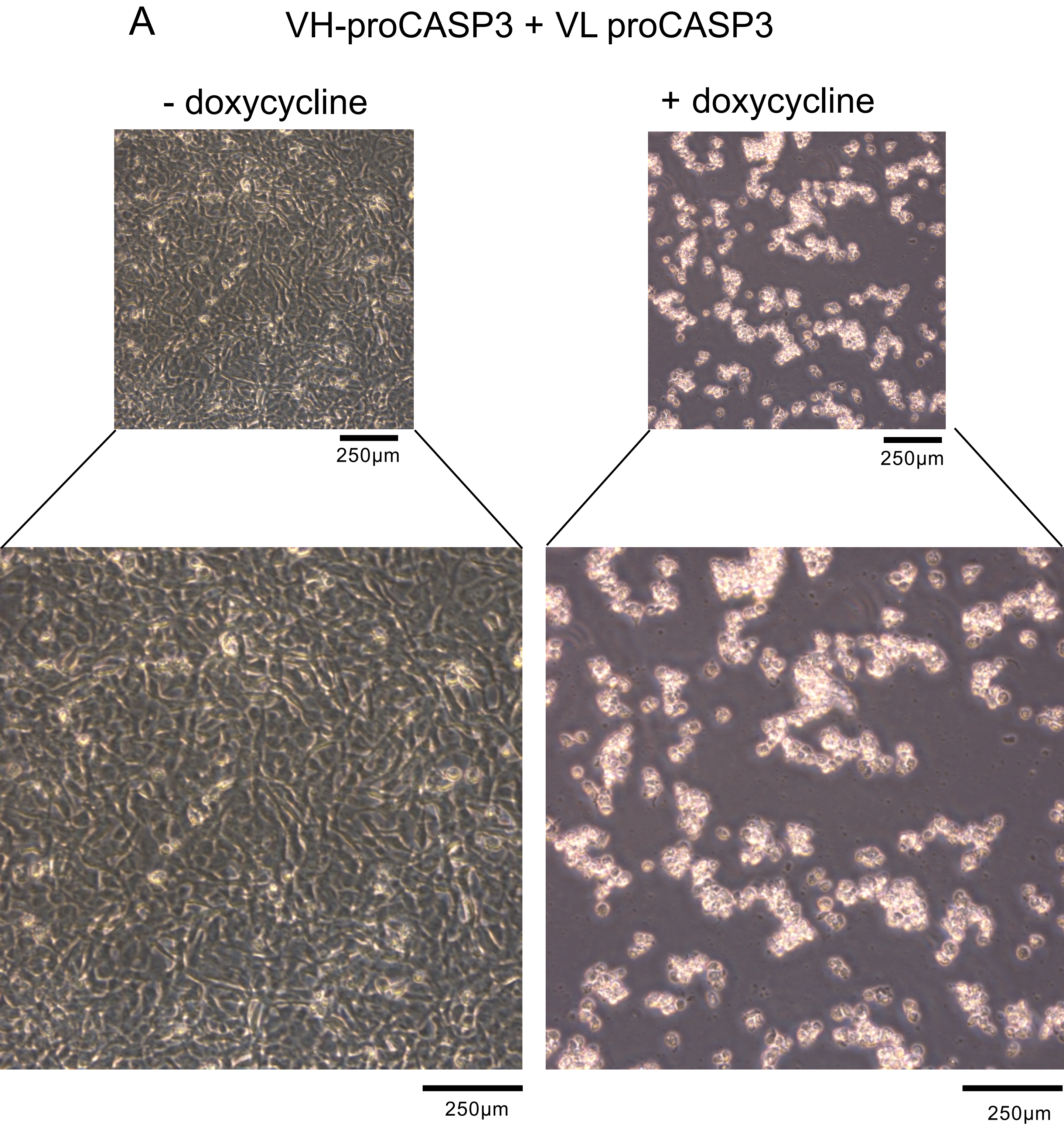

A
VH-proCASP3 + VL proCASP3
- doxycycline
+ doxycycline
250μm
250μm
250μm
250μm

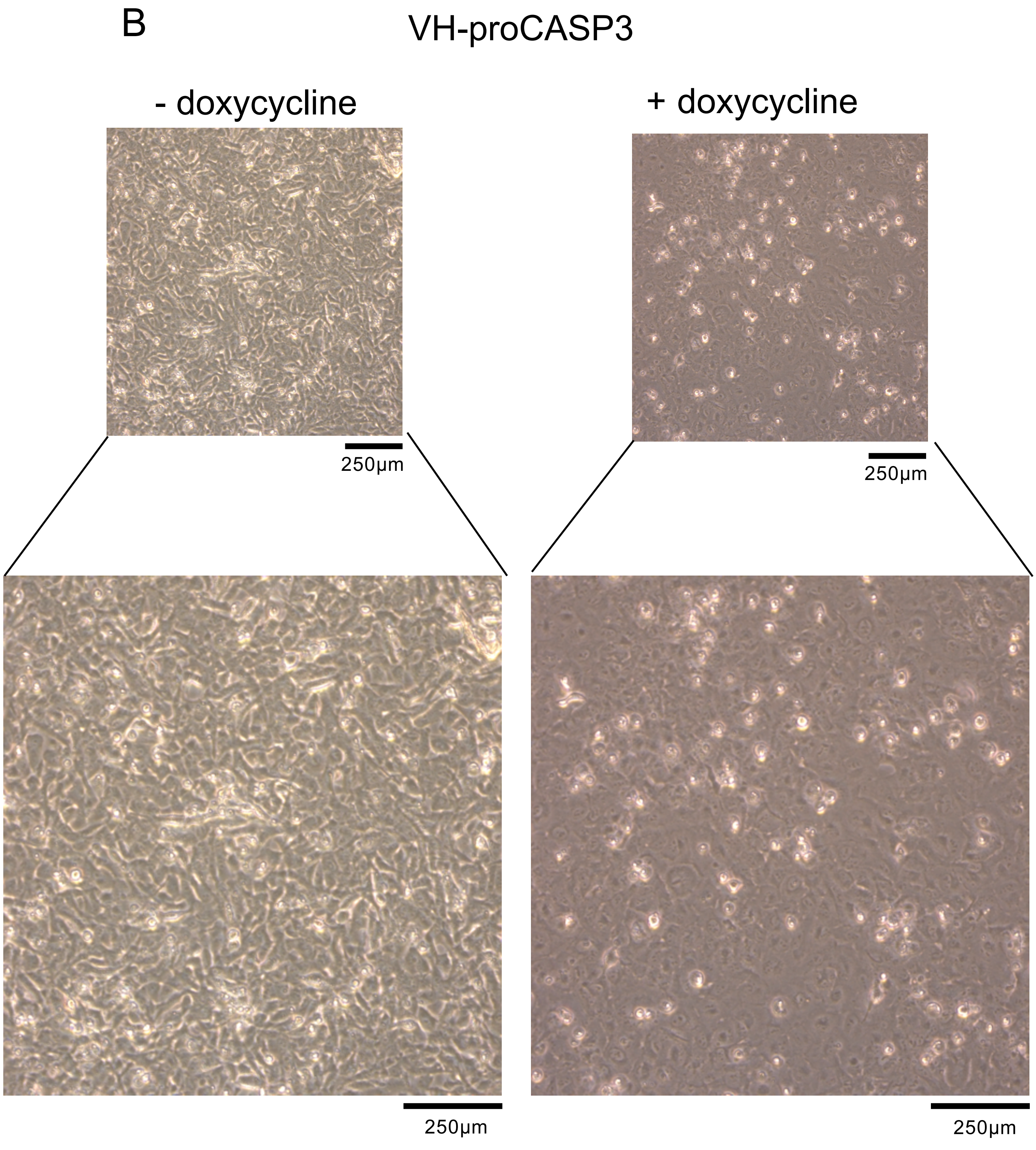
B
VH-proCASP3
- doxycycline
+ doxycycline
250μm
250μm
250μm
250μm

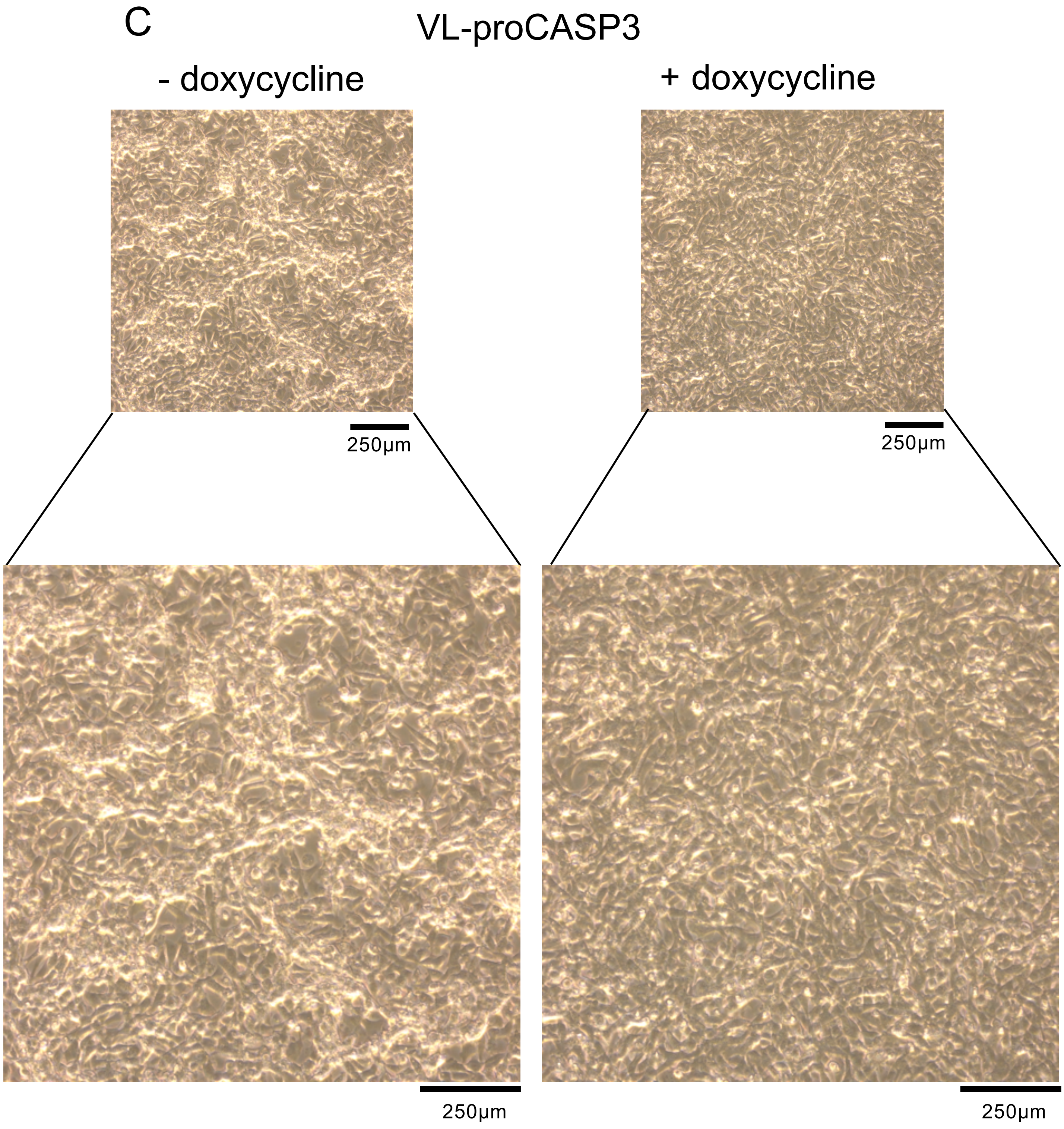

Supplementary Figure 2. Light microscope images (10x) of cell lines after induction of the VH-proCASP3 and VL-proCASP3 protein.
Morphological comparison of cells cultured with or without doxycycline-containing medium for 72 hours. Representative bright field images of the three original AIDA construct clones taken with a 10x objective lens. Below each image is an expanded version.

A: a VH-proCASP3+VL-proCASP3 clone
B: a VH-proCASP3
C: a VL-proCASP3 clone
Scale bars are indicated.

Figure S3

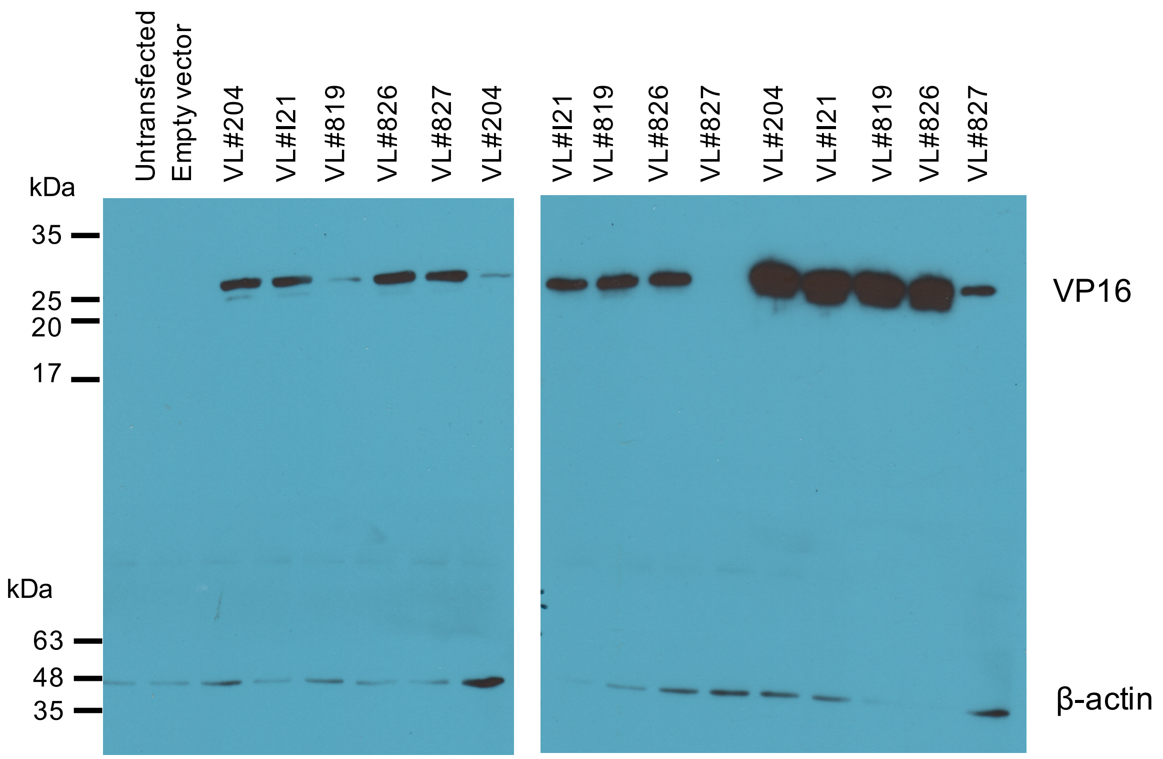

Supplementary Figure 3: VH and VL iDAb bind to RAS in tertiary complex

HEK293T cells transiently co-transfected with plasmids encoding mutant KRAS^{G12V} antigen, various indicated VH-VP16 and VL-Gal4DBD in addition to Renilla luciferase and a Firefly luciferase reporter plasmid pG5-Luc. The expressed anti-RAS VH-VP16 fusion proteins were wild type VH, lower affinity, dematured (called VHdm) and mutant (called VHmut) and represented by red, blue and green respectively. Transfections included the anti-RAS VL (VL204) and four non-relevant VLs; VLI21 (no specific) and the anti-LMO2 VL domains VL819, VL826 and VL827. The cells were lysed for Western blot and10μg total protein loaded per lane of two SDS-polyacrylamide gels. The two gels were placed on a single membrane for protein transfer and the membrane probed with an anti-VP16 antibody to detect VH (top part) and an anti-β-actin antibody as a loading control (bottom half)

Figure S4

A

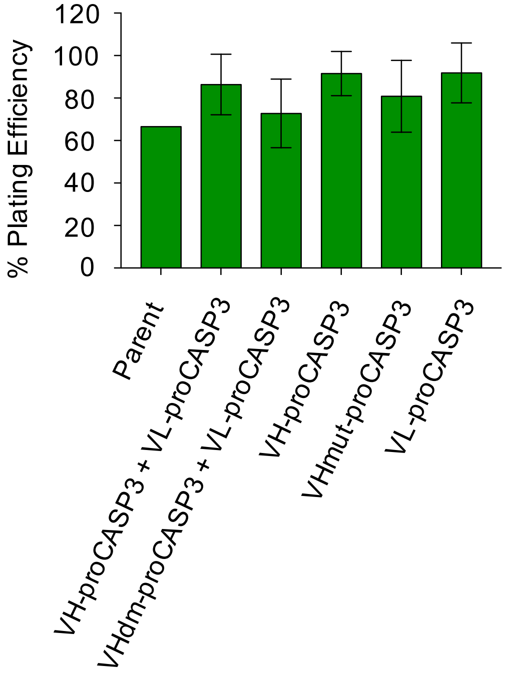

B

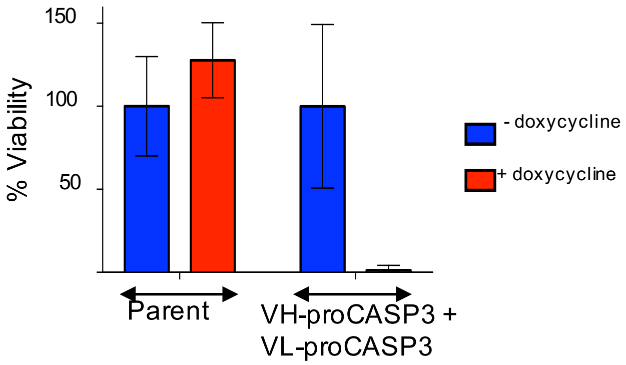

Supplementary Figure 4: Minimal clonogenic assay plating efficiency & spheroid viability

A. Histogram showing the plating efficiency data from the minimal clonogenic assay. After 9 days in culture without doxycycline, the number of foci ≥10 cells were counted across all replicates of each plating density. The percent efficiency of each indicated clone type to form colonies was calculated. Error bars represent standard deviation from the mean. The plating efficiencies were used to calculate the respective clone-type surviving fraction (shown in Figure 3).

B. Tumour spheroids. formed from the parental line and one VH-proCASP3+VL-proCASP3 clone, were treated with and without doxycycline for 18 days. Viability assessment of the clone set using the Prestoblue (Invitrogen) resazurin-based reagent. Spheroids were incubated with (red bars) and without (blue bars) doxycycline over the course of the experiment and stained with reagent for twenty minutes. Fluorescence was measured at 544nm excitation and 590nm emission. The no doxycycline-treated wells were normalized to an average 100% fluorescence to allow comparison between clones and clone types. The averaged doxycycline-treated readings were converted to percentage based on no added doxycycline, averaged measurements. Number of wells analyzed are Parent no doxycycline, n = 12; Parent with doxycycline, n = 4; VH-proCASP3+VL-proCASP3 no doxycycline, n = 11; VH-proCASP3+VL-proCASP3 with doxycycline n = 12.
